# The Role of Renal Replacement Therapy in the Management of Pharmacologic Poisonings

**DOI:** 10.1155/2016/3047329

**Published:** 2016-11-30

**Authors:** Aibek E. Mirrakhimov, Aram Barbaryan, Adam Gray, Taha Ayach

**Affiliations:** ^1^Department of Medicine, University of Kentucky College of Medicine, Lexington, KY, USA; ^2^Department of Internal Medicine, University of Kansas Medical Center, Kansas City, KS, USA; ^3^Division of Nephrology, Bone and Mineral Metabolism, University of Kentucky College of Medicine, Lexington, KY, USA

## Abstract

Pharmacologic toxicities are common and range from mild to life-threatening. The aim of this study is to review and update the data on the role of renal replacement therapy (RRT) in the management of various pharmacologic poisonings. We aim to provide a focused review on the role of RRT in the management of pharmacological toxicities. Relevant publications were searched in MEDLINE with the following search terms alone or in combination: pharmacologic toxicity, hemodialysis, hemofiltration, renal replacement therapy, toxicology, poisonings, critical illness, and intensive care. The studies showed that a pharmacologic substance should meet several prerequisites to be deemed dialyzable. These variables include having a low molecular weight (<500 Da) and low degree of protein binding (<80%), being water-soluble, and having a low volume of distribution (<1 L/kg). RRT should be strongly considered in critically ill patients presenting with toxic alcohol ingestion, salicylate overdose, severe valproic acid toxicity, metformin overdose, and lithium poisoning. The role of RRT in other pharmacologic toxicities is less certain and should be considered on a case-by-case basis.

## 1. Introduction

Pharmacological substances carry an intrinsic risk of toxicity as the result of either idiosyncrasy or overdose. For example, there were 2,188,013 cases of human exposures to various toxic substances resulting in 20,749 cases of serious adverse reactions and 1,552 deaths in 2013 [1]. In such cases, hemodialysis was used in more than 2,290 cases [2]. In the year 2014, pharmaceutical toxicities were responsible for 61.4% of cases and nonpharmacological exposures accounted for 14.1% of registered cases in 2014 [2].

The goal of this article is to review the data and evidence on the use of RRT in the management of certain pharmacologic overdoses. First, we review and discuss the different factors that would affect dialyzability of drugs and toxins. Second, we discuss different extracorporeal treatment modalities with focus on hemodialysis and hemofiltration treatments. Third, we review the role of RRT in the management of specific drugs and poisons including toxic alcohols, salicylate, lithium, metformin, valproic acid, and dabigatran. Lastly, we discuss the role of RRT in the management of less common miscellaneous cases of intoxication.

It is important to mention that the management of the toxicities mentioned above is complex and usually requires measures in addition to dialysis.

## 2. Removal of Drugs and Toxins by Extracorporeal Therapies

The use of extracorporeal techniques to remove toxins is justified if there is an indication of severe toxicity. The extent to which a drug is affected by extracorporeal therapies is determined primarily by several physicochemical characteristics of the drug which are summarized in Table 1. These include molecular size, protein binding, volume of distribution, water solubility, and endogenous clearance. In addition to these properties of the drug, technical aspects of the procedure may also determine the extent to which a drug is removed [3, 4].

### 2.1. Molecular Weight

Dialysis is dependent upon the use of a synthetic dialytic membrane with fixed pore size. The movement of drugs or other solutes is largely determined by the size of these molecules in relation to the pore size of the membrane. As a general rule, smaller molecular weight substances will pass through the membrane more easily than larger molecular weight substances.

### 2.2. Protein Binding

Another important factor determining drug removal during dialysis is the concentration gradient of unbound (free) drug across the dialysis membrane. Because the primary binding proteins for most drugs (mainly albumin) are of large molecular size, the drug protein complex is often unable to cross the dialysis membrane. Drugs with a high degree of protein binding will have a low plasma concentration of unbound drug available for dialysis and therefore lower clearance.

### 2.3. Volume of Distribution

The efficacy of toxin removal is also influenced by its theoretical volume of distribution (VD). A drug with a large VD is distributed widely throughout tissues and is present in relatively small amounts in the blood. Factors that contribute to a large VD include a high degree of lipid solubility and low plasma protein binding. Drugs with a large volume of distribution (>1 L/kg) are likely to be minimally dialyzed.

### 2.4. Water Solubility

The dialyzate used for hemodialysis is an aqueous solution. In general, drugs with high water solubility will be dialyzed to a greater extent than those with high lipid solubility. Highly lipid-soluble drugs tend to be distributed throughout tissues, and therefore only a small fraction of the drug is present in plasma and is accessible for dialysis.

### 2.5. Endogenous Clearance

This includes renal and nonrenal (mainly hepatic) clearance of the drug. Dialysis will have a limited impact if the rate of drug removal is significantly faster by endogenous routes (>4 mL/Kg/min). It is generally accepted that use of extracorporeal treatment is justified, if at least 30% can be added to total body clearance by such treatment.

## 3. Extracorporeal Treatment Modalities

The extracorporeal techniques most frequently employed for the removal of toxins are intermittent hemodialysis, continuous renal replacement therapy, and hemoperfusion. There are a few reports on the use of molecular adsorbent recirculating system (MARS) in poisoning, specifically for those toxins that are strongly protein bound; however, the use of MARS is limited by its availability, technical applicability, and high costs.

### 3.1. Intermittent Hemodialysis

During hemodialysis (HD), toxins and other solutes are cleared from the blood by diffusion against a steep concentration gradient through a semipermeable membrane into dialyzate. In addition to its specific properties (Table 1), the clearance of a toxic substance during HD depends also on membrane surface area and type, as well as on blood and dialyzate flow rates. HD comes in standard as well as high-efficiency or high-flux modalities. The major difference is the pore size of the membrane, the type of membrane, and the amount of dialyzate flow that occurs. Increasing blood and dialyzate flow rates can increase the concentration gradient between blood and dialyzate, thus optimizing the rates of diffusion and elimination. Clearances can also be enhanced by increasing dialyzer efficiency or membrane surface area. Larger-solute removal can be enhanced by increasing dialyzer flux when intermittent HD is used (for toxins >500 d and up to 10,000 d) or by switching to hemofiltration, which is usually applied continuously as discussed later.

The major drawback of HD is the risk of rebound toxicity after cessation of the treatment, due to redistribution of the toxin between body compartments. Extending the HD session beyond 4 hours can to some extent ameliorate rebound; however, this may not be easily feasible. An alternative or adjunctive solution is to increase dialysis session frequency or switch to continuous therapy after initial HD treatment specifically for substances with higher volume of distribution.

Intermittent HD is usually the first-choice extracorporeal modality because of its common availability, the rapidity of toxin removal, and the low molecular weight of the common agents of poisoning [5].

### 3.2. Continuous Renal Replacement Therapy (CRRT)

The use of CRRT has become common practice in Intensive Care Unit (ICU) settings during the last 2 decades for treatment of acute kidney injury. The term CRRT is commonly used to describe all continuous modalities of hemofiltration. Continuous venovenous hemofiltration (CVVH) is the most commonly used of the CRRT modalities, where dialysis occurs by convective transport. In continuous venovenous hemodiafiltration (CVVHDF), diffusive transport of molecules is combined with convective removal in order to mainly improve the clearance of small solutes [6].

The main advantage of CRRT is its applicability in hemodynamically unstable patients. It can be easily set up and run by regular ICU staff, thereby avoiding the need for specially trained dialysis nurses and technicians. The membranes used in CRRT are typically more permeable compared to standard intermittent HD membranes. Most high-flux HD membranes allow for the clearance of molecules up to 10,000 Da. CRRT membranes allow for the clearance of molecules as large as 20,000–40,000 Da and therefore would be the preferred modality for larger toxins removal. Another advantage of CRRT is the ability to avoid rebound of toxins removed from intravascular space, due to continuous nature of the procedure and slower rate of clearance, leading to less dramatic decreases in plasma drug levels and slower reequilibration of toxins between intracellular and intravascular spaces [5, 7].

Although CRRT gives better longer-term solute clearances (over the course of several days), it is less efficient in the short term and does not provide the rapidity of elimination afforded by intermittent HD when minimizing toxin exposure is a high priority. Other disadvantages of CRRT include the requirement for intensive anticoagulation which can place a patient at risk for bleeding and it is more associated with electrolyte disturbances. Finally, CRRT is not available at many smaller hospitals, possibly due to high equipment, training, and staffing costs [4, 8].

There are abundant case reports as well as a few small case series in the medical literature documenting the use of CRRT in the treatment of poisonings, but specific techniques and the clinical outcomes vary considerably. Therefore, one cannot draw definitive conclusions regarding benefit. Some patients, particularly those who are hemodynamically unstable and are not candidates for conventional HD, may warrant a trial of CRRT. If it is logistically possible, an ideal combination may be initial use of intermittent HD for rapid reduction of toxin levels followed by continuous therapy to ameliorate any postdialysis rebound when this is predicted. Controlled trials to better clarify the role of CRRT in treatment of poisonings would be beneficial, though such studies would be extremely difficult to conduct in this field [8].

### 3.3. Hemoperfusion

Hemoperfusion consists of the passage of anticoagulated blood through a cartridge containing an adsorbent material such as activated charcoal or a resin. In order to be removed by hemoperfusion, the toxic substance must have binding affinity to the sorbent in the cartridge and a low volume of distribution (Table 1). Water-soluble and lipid-soluble substances with molecular weights ranging from 100 to 40,000 daltons are well adsorbed with hemoperfusion.

In general, hemoperfusion is preferred to hemodialysis for the removal of chemicals that are lipid-soluble or are highly protein bound. However, the advantage of hemoperfusion over HD has lessened with the advent of high-flux dialysis membranes. Additionally, there is generally greater expertise and availability with respect to hemodialysis than hemoperfusion [42].

## 4. Renal Replacement Therapy in the Management of Specific Pharmacologic Poisonings

### 4.1. Toxic Alcohol Ingestion

Methanol, ethylene glycol, diethylene glycol, and isopropyl alcohol (also known as isopropanol) are alcohols commonly used in household solutions such as various cleaners, disinfectants, solvents, and antifreeze solutions as well as machine fluids [43–45]. There were 52,430 exposures to alcohols resulting in 174 fatalities in 2013 [46]. The vast majority of methanol, ethylene glycol, and isopropyl alcohol toxicities arise either as a result of suicidal attempts or after drinking the toxic alcohol as a substitute for ethanol [43]. However, the vast majority of diethylene glycol toxicities are the result of the introduction of diethylene glycol into various pharmacologic substances as a substitution for more expensive and less toxic substances [45].

To understand the basic pathogenesis of methanol, ethylene glycol, diethylene glycol, and isopropyl alcohol toxicities, it is important to briefly review the metabolism in vivo. When ingested, both methanol and ethylene glycol undergo an initial biochemical reaction catalyzed by alcohol dehydrogenase (the same enzyme metabolizing ethanol), which converts the parent alcohol into formaldehyde and glycolaldehyde, respectively. The final products of methanol and ethylene glycol metabolism are formic acid and oxalic acid, respectively [43]. The metabolism of methanol and ethylene glycol disrupts cellular energy metabolism leading to cellular damage [47, 48]. These end products result in classic features of toxicity such as retinal toxicity caused by methanol and renal injury mediated by oxalic acid.

The first step of diethylene glycol metabolism also involves the alcohol dehydrogenase enzyme which converts diethylene glycol into 2-hydroxyethoxyacetaldehyde [45]. Aldehyde dehydrogenase enzyme, in turn, converts 2-hydroxyethoxyacetaldehyde into 2-hydroxyethoxyacetic acid. The pathogenesis of diethylene glycol toxicity was first believed to involve the in vivo formation of ethylene glycol as the result of metabolism. However, further animal studies showed that the major toxic metabolite is 2-hydroxyethoxyacetic acid and that the metabolic conversion of diethylene glycol into ethylene glycol does not occur in vivo [45].

The first step of isopropyl alcohol in vivo metabolism also involves the enzyme alcohol dehydrogenase, which converts it into acetone. Acetone, in turn, undergoes several intermediate metabolic steps with the end product being glucose [44]. It is important to note that, in the vast majority of cases, isopropyl alcohol appears to be less toxic than methanol and ethylene glycol which are associated with greater toxicities and mortality rates [44, 47, 48].

**Figure 1:**
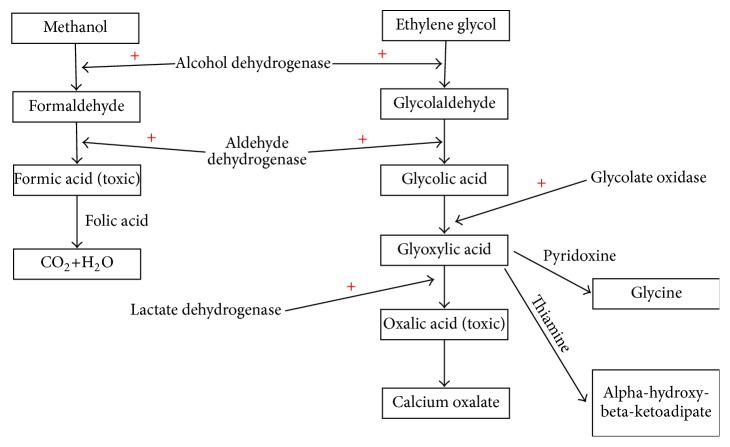
An overview of in vivo methanol and ethylene glycol metabolism.

**Figure 2:**
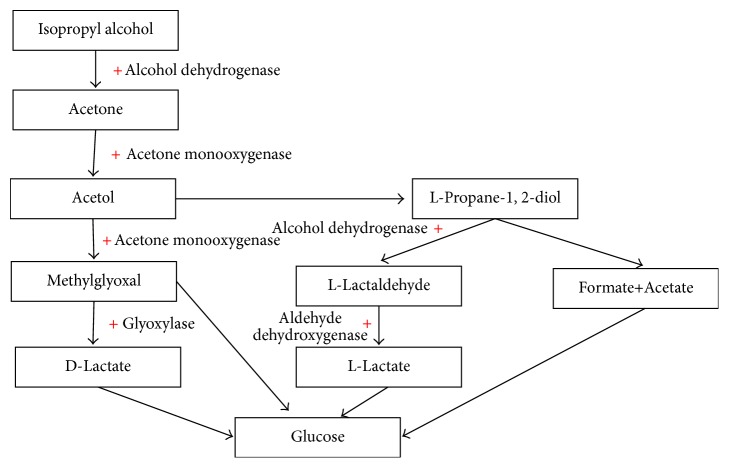
An overview of in vivo isopropyl alcohol metabolism.

The clinical presentations of methanol, ethylene glycol, and isopropyl alcohol overlap and include CNS depression, altered mental status, and seizures. Retinal toxicity and blindness are more specific for methanol intoxication, and acute kidney injury and hypocalcemia are more typical for ethylene glycol intoxication. Laboratory testing and diagnosis of methanol and ethylene glycol are based on the presence of a high anion gap metabolic acidosis, presence of a serum osmolal gap (a difference between measured osmolality and calculated osmolality ≥ 10), and measuring the levels of the toxic alcohols which is used for confirmation (typically these tests are not time sensitive, and treatment should not be withheld in any patient suspected of having toxic alcohol ingestion). Isopropyl alcohol laboratory findings include the presence of a high serum osmolal gap, presence of ketone bodies in the blood and/or urine (because of acetone), and typically the absence of a high anion gap metabolic acidosis. A brief overview of the in vivo metabolism of methanol and ethylene glycol is presented in Figure 1 and an overview of isopropyl alcohol metabolism is presented in Figure 2.

The management of methanol and ethylene glycol poisoning includes supportive care, respiratory support if needed (mechanical ventilation), the use of cofactors to stimulate formation of less toxic metabolites (see Figures 1 and 2), and the use of either an alcohol dehydrogenase inhibitor (fomepizole) or ethanol, which work by displacing the toxic alcohol and preventing it from being metabolized by alcohol dehydrogenase. Ethanol is less desirable and should only be used in cases of fomepizole unavailability. It is important to remember that fomepizole and ethanol have no effect on the metabolism and clearance of toxic metabolites such as formic acid and glycolic acid. Therefore, inhibition of alcohol dehydrogenase will not translate into an improved outcome once the parent alcohol has been metabolized.

RRT should be considered in cases of ongoing hemodynamic instability despite appropriate management and especially in the presence of severe metabolic acidosis, acute kidney injury, and target organ damage (retinal toxicity in methanol and acute kidney injury in ethylene glycol toxicity) [45, 47]. The small size, low VD, and low protein binding for these alcohols make them readily dialyzable, making standard hemodialysis the first-line therapy for extracorporeal elimination except in cases where hemodialysis is not available or in the setting of significant hemodynamic compromise where CRRT would be indicated [8]. Consensus guidelines recommend hemodialysis when the levels of parent alcohols exceed 50 mg/dL, although some patients without evidence of target organ damage, acute kidney injury, and metabolic acidosis may be managed without hemodialysis. Hemodialysis should also be considered in patients with ethylene glycol poisoning that have a persistent hyperosmolar state (despite appropriate management) [49] and levels of glycolic acid above 8 mmol/L [50]. It is important to mention that, in cases of methanol poisoning, hemodialysis enhances the clearance of methanol (the endogenous clearance of methanol is slow after alcohol dehydrogenase inhibition) [48], but it only marginally increases the clearance of formic acid [9]. Also, hemodialysis may be less costly than therapy with fomepizole; however, it is essential to remember that hemodialysis is associated with more complications and should be limited to patients with clear indications [51]. End goals of hemodialysis in these patients should be normalization of acid base status, resolution of hyperosmolar states, and a decreased blood level of parent toxic alcohols (less than 25 mg/dL). Redistribution of methanol and ethylene glycol can occur after hemodialysis and the serum electrolytes, osmolality, and acid base status should be monitored for additional 12–36 hours after the last hemodialysis treatment [47, 48].

Literature on the role of hemodialysis in the management of diethylene glycol is scant [45], and it is unclear whether the active toxic metabolite is removed by hemodialysis. Nevertheless, hemodialysis should be considered in patients with progressive clinical deterioration despite appropriate care and persistent high anion gap metabolic acidosis.

In cases of isopropyl alcohol toxicity, the role of hemodialysis is less clear [44]. Isopropyl alcohol intoxication generally has a more favorable outcome compared to methanol and ethylene glycol poisonings and the vast majority of patients will improve with supportive therapy and alcohol dehydrogenase inhibition. In the rare patient with hemodynamic instability and an isopropyl alcohol level above 4,000 mg/dL (which is usually due to a massive ingestion), hemodialysis may be considered.

When considering renal replacement therapy in these patients, it is important to note that both fomepizole and ethanol are cleared by hemodialysis and the doses of fomepizole and ethanol should be adjusted accordingly.

An overview of the clinical presentations, major laboratory findings, general principles of management, and indications for hemodialysis among patients with toxic alcohol ingestion is presented in Table 2. A summary of toxic alcohol pharmacokinetics and the utility of hemodialysis is presented in Table 3.

### 4.2. Salicylate Toxicity

Salicylates are a group of pharmacologic agents which includes aspirin, bismuth salicylate, and local skin preparations such as salicylic acid and methyl salicylate (topical preparations occasionally cause toxicity if used excessively or in patients with skin damage leading to increased absorption) [10, 52, 53].

Analgesics including aspirin are the most common etiology of all drug poisonings in the USA, and salicylate poisoning caused 34 out of 2,113 deaths due to poisonings reported in 2013 [51]. The major mechanism of action of aspirin is via inhibition of cyclooxygenase enzyme resulting in decreased production of thromboxane A2 and various prostaglandins [11]. However, with higher dosages, other biochemical alterations may occur such as uncoupling of oxidative phosphorylation in the electron transport chain resulting in heat release and stimulation of the respiratory center in the medulla. A decrease in blood pH will favor formation of lipid-soluble salicylic acid which easily penetrates the blood brain barrier and undergoes renal reabsorption [54]. When used therapeutically, aspirin has a high degree of protein binding which significantly decreases in cases of overdose and poisoning.

Patients with salicylate toxicity typically present with tinnitus, gastrointestinal complications (nausea, vomiting, bleeding, and liver toxicity), hyperthermia (via uncoupling of oxidative phosphorylation), pulmonary edema, and a mixed acid-base disorder (high anion gap metabolic acidosis and respiratory alkalosis via stimulation of respiratory center in the brainstem) [54, 55].

Recent consensus panel guidelines on the management of severe salicylate toxicity recommend intermittent hemodialysis over other modalities of extracorporeal removal [12]. Hemodialysis should be strongly considered in patients with an altered mental status (which may be reflective of high salicylate content in the CNS), pulmonary edema, hypoxemia, fluid overload states or presence of a medical condition limiting the administration of sodium bicarbonate (such as congestive heart failure), presence of either acute or chronic kidney failure (since it will limit the amount of sodium bicarbonate administration and endogenous salicylate clearance), salicylate levels > 90 mg/dL in acute toxicity and normal renal function and levels > 80 mg/dL in acute toxicity, and impaired renal function and in cases of failure of appropriate management [12]. A summary of aspirin pharmacokinetics and the utility of hemodialysis is presented in Table 3.

### 4.3. Lithium Toxicity

Lithium has been used in the management of bipolar disorder since the nineteenth century [56]. The exact mechanism of action of lithium is not clear, but it may involve modulation of intracellular signaling pathways [57]. The major route of lithium elimination is renal excretion, but it is important to note that about 80% of filtered lithium is reabsorbed [13].

Lithium has a narrow therapeutic window and is associated with numerous side effects [57]. Several clinical scenarios of lithium toxicity can occur such as acute overdose in a suicidal patient, acute on chronic toxicity in patients taking lithium whose renal function has declined (e.g., patients with gastroenteritis, decreased oral intake, and patients concomitantly taking other medications such as nonsteroidal anti-inflammatory drugs and angiotensin converting enzyme inhibitors), and chronic toxicity in patients who slowly accumulate the medication and develop toxicity [14, 57]. Lithium was responsible for 6,610 cases of toxicities including 5 fatalities in the year 2013 [1].

Patients with chronic lithium poisoning typically develop nephrogenic diabetes insipidus and urinary concentrating defects, neurologic symptoms (ataxia, tremors, and altered mental status), hyperparathyroidism, hypothyroidism, and weight gain [14]. Patients with more acute presentations tend to have more pronounced gastrointestinal symptoms such as nausea, vomiting, diarrhea, cardiac arrhythmias, and neurologic symptoms.

Laboratory findings of acute lithium intoxication may include a negative anion gap and an osmolal gap [15]. Also it is important to assess kidney function since acute kidney insults often precipitate lithium toxicity.

The management of lithium intoxication includes stopping the offending medication, supportive care, and, in selected cases, renal replacement therapy such as hemodialysis [15]. Hemodialysis should be considered in patients with lithium levels > 4 mEq/L regardless of symptomatology and in patients with lithium levels > 2.5 mEq/L who either are symptomatic or have some clinical factors (advanced kidney disease and decompensated congestive heart failure) limiting the use of intravenous hydration. The end points of hemodialysis in patients with lithium toxicity are resolution of clinical symptoms of toxicity and lithium levels < 1 mEq/L [15]. However, it is important to monitor lithium levels after the cessation of hemodialysis, since lithium tissue stores can be redistributed into the bloodstream [15, 16]. Most cases of lithium intoxication treated with hemodialysis require at least a second session of hemodialysis following rebound. This rebound can be avoided by use of CRRT as described above preferably after the initial hemodialysis session for rapid reduction of lithium level [8, 15].

A summary of lithium pharmacokinetics and the utility of hemodialysis is presented in Table 3.

### 4.4. Valproic Acid Poisoning

Valproic acid is used for the management of epilepsy, bipolar disorder, migraine headaches, and peripheral neuropathy. The mechanism of action includes modulation of gamma aminobutyric acid activity and sodium channel blockade [17]. Valproic acid is a fatty acid and its toxicity is believed to involve the inhibition of mitochondrial beta oxidation [18]. Valproic acid has a favorable molecular weight and volume of distribution to be cleared by hemodialysis, though the degree of protein binding is high at therapeutic concentrations. However, the degree of protein binding decreases with extra therapeutic concentrations due to protein saturation, thus making it amenable for hemodialysis [19]. Valproic acid was responsible for 7,776 toxicities including 2 fatalities in the year 2013 [1].

Clinical manifestations may include altered mental status, tremors, myoclonus, hypotension, tachycardia, and respiratory depression [58]. Classic laboratory abnormalities include hyperammonemia, presence of an osmolal gap, hypernatremia, high anion gap metabolic acidosis, and elevated liver enzymes.

Management of acute valproic acid intoxication includes supportive care, administration of naloxone to help against respiratory depression, and antidote therapy with carnitine supplementation to offset the inhibitory effects on mitochondrial fatty acid oxidation. RRT should be strongly considered in patients with severe toxicity including those with cerebral edema (patients with papilledema, focal neurologic deficits, altered mental status, imaging findings, etc.) and hemodynamic instability and in those with valproic acid levels > 1300 mg/L. RRT should also be considered in patients with valproic acid levels > 900 mg/L, respiratory depression, hyperammonemia, and severe metabolic acidosis (pH < 7.1). Intermittent HD is the preferred modality of RRT in valproic acid poisoning. If hemodialysis is not available, then intermittent hemoperfusion or continuous renal replacement therapy is an acceptable alternative. The end point of hemodialysis includes clinical stabilization and valproic acid levels < 100 mg/L [19]. As in many other cases of intoxication, it is important to monitor valproic acid levels after the cessation of hemodialysis, since redistribution of the medication can cause reemergence of toxicity. A summary of valproic acid pharmacokinetics and the utility of hemodialysis is presented in Table 3.

### 4.5. Metformin Poisoning

Metformin is the most commonly used oral antihyperglycemic agent worldwide [58]. Besides treatment of type 2 diabetes mellitus and prediabetes, it is often used in the management of polycystic ovarian syndrome [59]. Metformin's mechanism of action includes decreased hepatic and intestinal gluconeogenesis, enhanced glucose utilization, and modulation of mitochondrial oxidation of fatty acids [20, 59]. The majority of overdose cases occur in patients with renal disease (either acute or chronic), advanced liver disease, and acute concurrent illness [20]. Metformin was responsible for 8,829 toxicities including 12 fatalities in the year 2013 [1].

The pharmacokinetics of metformin are generally favorable for hemodialysis and extracorporeal elimination such as a low molecular weight and minimal protein binding except with high volume of distribution [20, 21]. Clinical manifestations of metformin poisoning are nonspecific and may include gastrointestinal symptoms such as nausea, vomiting, diarrhea, abdominal pain, altered mental status, and hemodynamic instability. Laboratory features of metformin poisoning include a high anion gap metabolic acidosis due to accumulation of lactic acid. The initial mechanism of lactic acidosis involves modulation of gluconeogenesis [20]. However, later, hemodynamic deterioration may underlie the perpetuation of lactic acidosis.

Treatment of metformin poisoning includes supportive care and RRT. The low molecular weight, negligible plasma protein binding, and rapid transport of drug from cells to serum allow for drug removal by hemodialysis despite a relatively large VD. It is unclear whether hemodialysis improves mortality in patients with metformin poisoning. Furthermore, its efficacy may be suboptimal in patients who present after tissue redistribution occurs as that leads to a large volume of distribution. Nevertheless, hemodialysis should be strongly considered in patients with advanced renal failure, decompensated congestive heart failure, severe metabolic acidosis (pH < 7.1), and hemodynamic and clinical decline despite supportive care [20]. Whenever possible, prolonged sessions of hemodialysis should be undertaken; alternatively CRRT can be considered [22]. A summary of metformin pharmacokinetics and the utility of hemodialysis is presented in Table 3.

### 4.6. Dabigatran Poisoning

Dabigatran is a non-vitamin K oral anticoagulant used in the management of nonvalvular atrial fibrillation, venous thromboembolism, and postprocedural deep venous thrombosis prophylaxis [23]. Dabigatran represents an alternative to vitamin K antagonists in the vast majority of patients with the above-mentioned conditions. Dabigatran is predominantly excreted by the kidneys and is contraindicated in patients with advanced renal disease typically defined as creatinine clearance < 30 mL/min.

The major adverse effect related to the use of dabigatran is bleeding which may be minor or life-threating, such as in case of intracranial hemorrhage. Until recently, the management of bleeding in patients taking dabigatran was supportive [24]. However, recently, a monoclonal antibody, idarucizumab, has been shown to be effective in the management of patients with dabigatran related bleeding [25].

Before the availability of idarucizumab, RRT was used in the bleeding patient taking dabigatran. Several case reports highlighted the efficacy of RRT in the management of patients with dabigatran related bleeding [26]. In a small study, intermittent hemodialysis enhanced elimination of dabigatran more efficiently than CRRT, though dabigatran levels may rebound after cessation of hemodialysis via the effect of redistribution [27]. Dabigatran levels should be repeated and repeat hemodialysis should be considered in patients with a rebound increase in dabigatran concentration. Alternatively, longer duration hemodialysis sessions or CRRT may be considered specifically that most patients with dabigatran toxicity are critically ill with life-threatening bleeding or are in need for an emergent surgery, where CRRT would be more tolerated. However, it is important that the vast majority of patients with dabigatran poisoning do not have advanced renal disease and do not receive renal replacement therapy. Therefore, a dialysis vascular catheter must be inserted which may be difficult in an overanticoagulated patient and result in more bleeding and other complications. Thus, RRT should be considered in patients with severe bleeding and patients on dabigatran requiring emergent surgery when idarucizumab is not available. It is important to note that RRT does not have a role in the management of other non-vitamin K anticoagulants.

A summary of dabigatran pharmacokinetics and the utility of hemodialysis is presented in Table 3.

### 4.7. Miscellaneous Pharmacologic Poisonings

RRT has been effective in the management of various medication related toxicities [28–41, 60–66]. It is important to note that scientific data is limited to case reports and case series. RRT should be considered in patients with severe toxicity after failure of supportive care. A summary of some of these medications and the role of hemodialysis in the management of these toxicities is presented in Table 4.

## 5. Conclusion

The use of RRT should be considered in patients with toxic alcohol poisoning, salicylate toxicity, lithium overdose, and metformin poisoning as well as valproic acid toxicity. The role of RRT in the management of dabigatran toxicity is likely limited to cases with severe bleeding when idarucizumab is not available. RRT use should also be considered in the management of other drug toxicities on a case-by-case basis.

## Figures and Tables

**Table 1:** Optimal physicochemical properties for extracorporeal removal of drugs.

	Hemodialysis	Hemofiltration	Hemoperfusion
Molecular weight	<500 Da	<40 KDa	<40 KDa
Protein binding	Low (<80%)	Low	Low or high
Volume of distribution	<1 L/Kg	<1 L/Kg	<1 L/Kg
Solubility	Water	Water	Water or lipid
Endogenous clearance	<4 mL/Kg/min	<4 mL/Kg/min	<4 mL/Kg/min

**Table 2:** The features of toxic alcohol poisonings.

Type of toxic alcohol	Core clinical features	Core laboratory features	General principles of treatment	Indications for RRT
Methanol	CNS depression AMS Seizures Visual changes/retinal toxicity Hemodynamic instability	HAGMA High osmolal gap Elevated lactic acid (formic acid mediated inhibition of mitochondrial electron transport chain)	Supportive care Fomepizole Ethanol (if fomepizole is unavailable) Folic acid or folinic acid	pH < 7.3 Methanol level > 50 mg/dL Visual changes AKI Severe electrolyte derangements Hemodynamic instability and progression despite appropriate care

Ethylene glycol	CNS depression AMS Seizures AKI Calcium oxaluria	HAGMA High osmolal gap Hypocalcemia Electrolyte abnormalities AKI Calcium oxalate crystals in the urine Falsely elevated lactic acid (glycolic acid can be mistaken for lactic acid)	Supportive care Fomepizole Ethanol (if fomepizole is unavailable) Thiamine Pyridoxine	pH < 7.3 Ethylene glycol level > 50 mg/dL Glycolic acid level > 8 mmol/L Refractory hyperosmolarity AKI Severe electrolyte derangements Hemodynamic instability and progression despite appropriate care

Diethylene glycol	CNS depression AMS Seizures AKI Gastrointestinal symptoms Peripheral neuropathy	HAGMA High osmolal gap Elevated liver enzymes	Supportive care Fomepizole Ethanol (if fomepizole is unavailable) Thiamine Pyridoxine	Hemodynamic instability and progression despite appropriate care Persistent HAGMA

Isopropyl alcohol	CNS depression AMS Hemodynamic instability in advanced cases	High osmolal gap Increased ketones in the blood and urine Absence of HAGMA Falsely elevated creatinine (due to acetone cross reactivity)	Supportive care Fomepizole Ethanol (if fomepizole is unavailable)	Hemodynamic instability and progression despite appropriate care Isopropyl alcohol level > 4000 mg/dL

CNS: central nervous system.

AMS: altered mental status.

HAGMA: high anion gap metabolic acidosis.

AKI: acute kidney injury.

**Table 3:** Summary of pharmacological and clearance properties of some pharmacological substances^*∗*^.

Substance	Molecular weight (daltons)	Protein binding (%)	Volume of distribution (L/kg)	Metabolism and excretion (%)	Clearance without hemodialysis (mL/min)	Clearance with hemodialysis (mL/min)
Methanol	~32	Minimal	~0.6–0.77	~95 hepatic ~2.5 respiratory ~1 renal	~11.3	~125–215

Ethylene glycol	~62	Minimal	0.5–0.8	~80 hepatic ~20 renal	Up to 27	145–230

Diethylene glycol	~62	Minimal	~1	30–50 hepatic 50–70 renal	Unknown	Unknown

Isopropyl alcohol	~60	Minimal	~0.45–0.55	80 hepatic 20 renal	Unknown	~137 (isopropyl alcohol) ~165 (acetone)

Aspirin	~180	~49 (~90 with therapeutic use and ~30 in overdose)	~0.15	~80 hepatic ~20 renal	0.6–25	3–100

Lithium	~74	0	~0.3–1	>95 renal	20–40	70–170

Valproic acid	~144	~80–90 (continuously decreases with higher valproic acid concentrations)	~0.1–0.5	Predominantly hepatic	5–10	~50–90

Metformin	~129	Minimal	~1.1	>90 renal	~7	Up to 170

Dabigatran	471	~35	~0.85	>80 renal	Dependent on renal function	Decreases dabigatran concentration by at least 40%

^*∗*^Adapted from [5–20].

**Table 4:** Role of hemodialysis in the management of miscellaneous pharmacological poisonings.

Medication	Therapeutic use	Classic toxicity	Treatment	Efficacy of hemodialysis (HD)
Carbamazepine	Epilepsy Trigeminal neuralgia Bipolar disorder	Altered mental status Seizures Hemodynamic instability Arrhythmias	Supportive	May reduce carbamazepine level by about 50% [21–23]

Phenobarbital	Epilepsy	Altered mental status	Supportive	May reduce phenobarbital level by up to 59% after 4 h HD [24–26]

Phenytoin	Epilepsy Cardiac arrhythmias	Horizontal nystagmus Ataxia Altered mental status Arrhythmias Hypersensitivity reactions	Supportive	Should be considered in patients with severe poisoning not responding to supportive care [27]

Baclofen	Spasticity	Muscle hypotonia Altered mental status Hemodynamic instability	Supportive	Conventional HD can decrease the concentration by up to 79% [28–30]

Eptifibatide	Antiplatelet agent Acute coronary syndrome	Bleeding	Supportive Platelet transfusion	Limited to patients with renal failure experiencing ongoing severe bleeding not responding to supportive care [31]

Diltiazem Atenolol	Hypertension Cardiac arrhythmias	Bradycardia Hemodynamic instability	Supportive	HD may be considered in unstable patient with renal failure not responding to supportive care [32, 33]

Lisinopril	Hypertension Heart failure Renal disease	Acute kidney injury Hyperkalemia Hemodynamic instability Angioedema	Supportive	HD may be considered in unstable patient with renal failure not responding to supportive care [34]

Theophylline	Obstructive pulmonary disease	Arrhythmias Altered mental status Seizures	Supportive	HD may be considered in unstable patient with renal failure not responding to supportive care [35, 36]

Cefepime	Antibiotic	Neurotoxicity Altered mental status	Supportive	HD may be considered in unstable patient with renal failure not responding to supportive care [37]

Metronidazole	Antibiotic	Altered mental status Seizures Neuropathy Gastrointestinal symptoms	Supportive	HD should be considered in patient with metronidazole overdose and renal failure [38]

Dapsone	Antibiotic	Hypersensitivity reactions Methemoglobinemia	Supportive Methylene blue	HD should be considered in patients not responding to conventional therapy [39]

Isoniazid	Antibiotic	Neurotoxicity Seizures Liver toxicity	Supportive Pyridoxine	HD should be considered in patients not responding to conventional therapy [40]

Acetaminophen	Analgesic	Liver failure	Supportive N-Acetylcysteine Liver transplant	HD may be considered in unstable patients with metabolic acidosis [41]

## Additional Points

Why is this topic important? It critically reviews the scientific literature on the management of pharmacological toxidromes. What does this review attempt to show? It attempts to show the role of hemodialysis in the management of pharmacological poisonings. What are the key findings? Hemodialysis should be strongly considered in patients with toxic alcohol ingestion, salicylate overdose, severe valproic acid toxicity, metformin overdose, and lithium poisoning. Hemodialysis should be considered on case-by-case basis in the management of toxidromes secondary to other pharmacological agents. How is patient care impacted? Hemodialysis in the management of various pharmacological toxidromes which can be fatal should be considered. Early consultation with nephrologist regarding hemodialysis initiation is advised.
